# On the origin of the reward function: Exploring the role of conditioned reinforcement and social learning

**DOI:** 10.3758/s13423-025-02813-3

**Published:** 2025-12-18

**Authors:** Francesco Rigoli

**Affiliations:** Department of Psychology, City St George’s, University of London, Northampton Square, London, EC1V 0HB UK

**Keywords:** Reward function, Goal-directed, Conditioned reinforcement, Imitation, Value, Social learning

## Abstract

Influential cognitive science theories postulate that decision-making is based on treating expected outcomes as incentives according to a reward function. Yet a systematic analysis of the learning processes that determine the reward function remains to be carried out. The paper fills this gap by examining the contribution of two fundamental learning processes: *conditioned reinforcement*, occurring either via direct or via vicarious experience, and *imitative incentive learning*, at play when an agent appropriates the incentives sought by another individual. From an evolutionary perspective, the two processes appear to be adaptive insofar as conditioned reinforcement might have evolved to simplify decision-making, while imitative incentive learning might have arisen to harness the full potential of social learning and to facilitate cooperation. The paper contributes to research on decision-making by offering a detailed analysis of the learning mechanisms that drive acquisition of the reward function.

## Introduction

**Fig. 1:**
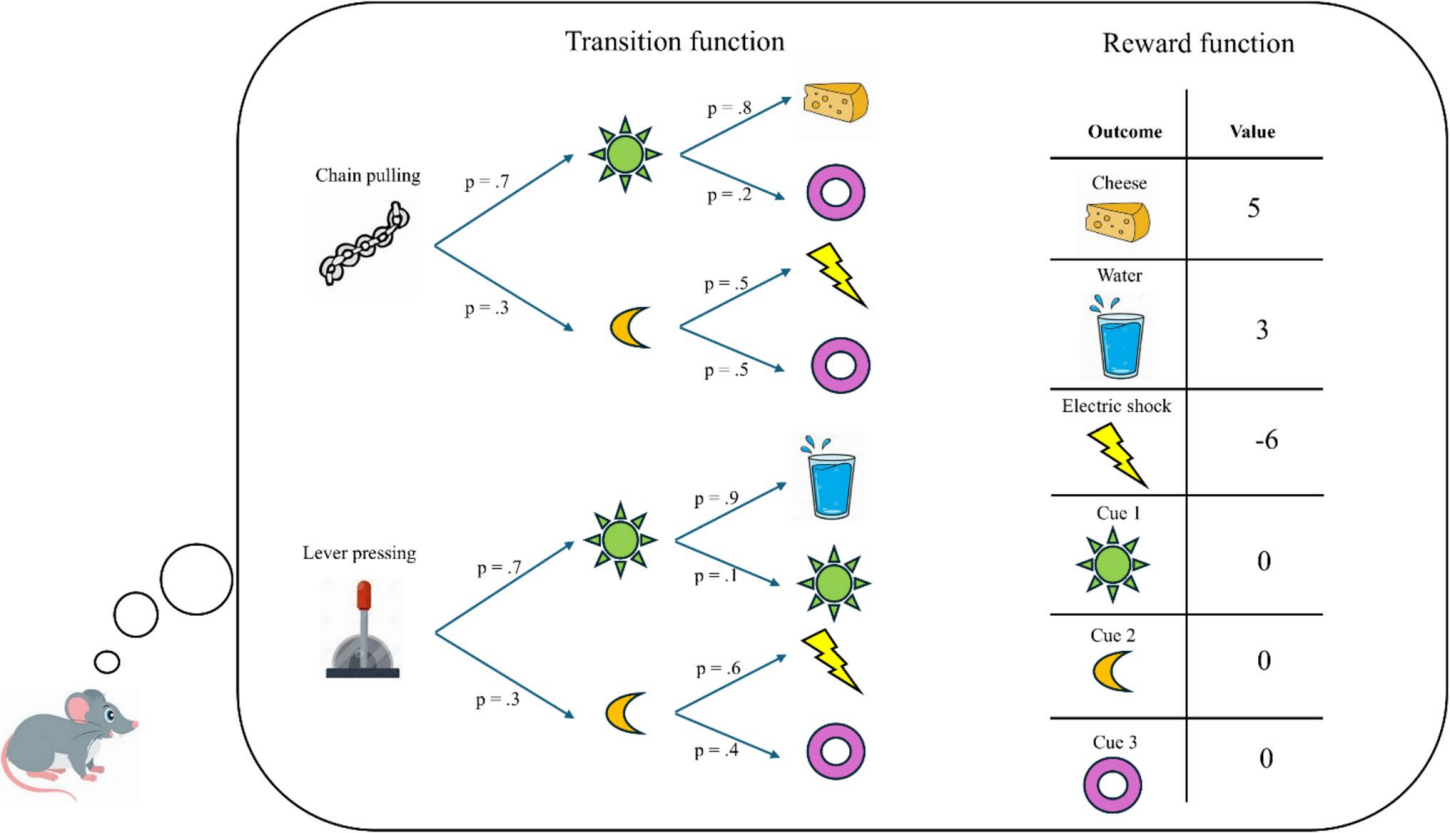
Schematic illustration of decision-making, describing a scenario where a mouse has to make a choice between two available options—chain pulling and lever pressing. Decision-making is based on integrating two kinds of beliefs: the *transition function*, representing beliefs about action-outcome contingencies, and the *reward function*, representing beliefs about the value of outcomes. (Color figure online)

Influential cognitive science theories presuppose that human decision-making is, at least to some extent, goal-directed, or, employing the terminology of reinforcement learning, model-based (for recent versions of this notion, see Castegnetti et al., 2021; Chu et al., 2024; Cushman & Morris, 2015; Frömer et al., 2019; Juechems et al., 2019; Juechems & Summerfield, 2019; Keramati & Gutkin, 2014; O’Reilly, 2020). The idea is that, at any point in time, our brain is capable to represent a decision tree by simulating different courses of action (Fig. 1) (Bellman, 1957; Sniedovich, 1991). By representing the consequences of a chain of actions, the decision tree can extend deep into the future. Moreover, by postulating some uncertainty about which outcome is produced by any action, it can be probabilistic. Any decision tree is constructed based on two sources of knowledge: the *transition function* and the *reward function* (Sutton & Barto, 1999). The former corresponds to beliefs about action-outcome contingencies, and it can be expressed verbally by the proposition “if state *x* occurs at time *t*, then action *a* leads to state *y* at time *t* + 1 with probability *p.*” For example, the transition function captures the belief that if an actor is in the kitchen now, then opening the fridge leads to a chocolate bar with probability 0.9. Intuitively, the transition function describes beliefs about a map of the environment and about the action rules at play to navigate it. The other element of the decision tree is the reward function. To each outcome represented within the decision tree, the reward function attaches a number which describes how desirable (when the number is positive) or undesirable (when it is negative) the outcome is. For example, the fridge may be linked with indifference, thus being attached a value of zero, while the chocolate, being highly desirable, may be linked with a positive value of 10. Combining the transition function with the reward function is, within this framework, what determines choice behavior.^1^

Based on this picture, a critical question is where the reward function comes from. For example, why, during decision-making, the reward function assigns a value of zero to the fridge and a positive value to the chocolate bar? Before addressing this question, a note on terminology is warranted. We shall describe the reward function using the following terms. The term *reward* will refer to outcomes which, according to the reward function, are linked with a positive number. The term *punishment* will refer to outcomes associated with a negative number. Collectively, rewards and punishments will be referred as *incentives*. Finally, outcomes linked with a value of zero will be referred as *neutral outcomes*.

Armed with these definitions, we can reformulate the above question as follows: why, during decision-making, are certain outcomes viewed by the brain as incentives, while other outcomes are viewed as neutral? With regard to this question, contemporary theories of decision-making (Castegnetti et al., 2021; Chu et al., 2024; Cushman & Morris, 2015; Frömer et al., 2019; Juechems & Summerfield, 2019; Juechems et al., 2019; Keramati & Gutkin, 2014; O’Reilly, 2020) typically assume that, when the organism is in the appropriate motivational state (e.g., hunger), certain stimuli (e.g., food) are treated as incentives by evolutionary design. Is the reward function predicated solely in this way, that is, based on outcomes that are inherently treated as incentives by evolution—we shall call these *innate incentives*? Or, rather, can outcomes which are neutral at the outset become incentives after learning—we shall call these *acquired incentives*? This question has rarely been investigated explicitly.

The paper overviews the empirical literature that is relevant to assess whether, during decision-making, the reward function includes also acquired incentives alongside innate ones. Our examination documents at least two empirical phenomena which reveal the existence of acquired incentives at play during decision-making. The first of such phenomena is *conditioned reinforcement* (Hendry, 1969; Wike, 1966; Williams, 1994a, 1994b). The second one is a specific type of social learning that we label *imitative incentive learning* (Bandura, 1977; Fryling et al., 2011; Greer et al., 2006). On this basis, the purpose of the present paper is to carry out a systematic exploration of how the reward function is learnt, with a particular focus on two major processes involved: conditioned reinforcement and social learning. To this aim, the paper will review, and critically evaluate, the previous empirical literature that speaks to the question of how conditioned reinforcement and social learning contribute to shape the reward function. Note that, rather than reviewing the literature on conditioned reinforcement and social learning in a systematic fashion, the paper will specifically analyze aspects that are relevant to assess how the reward function is acquired. This represents the major novelty of the paper, as previous investigations of conditioned reinforcement and social learning have never examined how these processes contribute to shaping the reward function. As we shall see, our investigation highlights questions that can be answered already in light of available empirical evidence alongside questions that remain open.

Before proceeding with our analysis, we believe it is important to clarify further the specific question addressed in the paper. This can be elucidated by considering the distinction between *means* and *ends*. In the context of the reward function, an outcome linked with a non-zero value can be considered to be an end (i.e., an approach end in the case of reward, and an avoidance end in the case of punishment) since it is valuable in and of itself.^2^ An outcome linked with a value of zero, by contrast, may sometimes be a means (when it is useful to achieve an end), but it is never an end in and of itself, since it is not valuable as such. Distinguishing between ends and means is paramount for our analysis, since we are interested in pinpointing the specific processes whereby ends, and not means, are learnt. On this basis, the question investigated here can be formulated as follows: what is the origin of human ends? And how are these ends learnt?^3^

To tackle the paper’s question, it is useful to start by overviewing the innate factors upon which the reward function is grounded. This will allow us to explore how learning processes build on these innate factors.

## Innate incentives

Psychological and biological theories commonly assume that, for any animal species, there are some outcomes which are inherently good or bad for survival (Toates, 1986). Examples are meat and grass for carnivorous and herbivorous animals, respectively, as well as painful experiences, such as when a limb is too close to fire. The assumption is that, when facing these outcomes, evolution has developed an innate motivational tendency towards them. Specifically, when approaching an outcome promotes survival for the species, such as in the case of food, the outcome works as an *innate reward*, implying that it will be associated with a positive number in the reward function. By contrast, when avoiding an outcome promotes survival, such as in the case of painful experiences, the outcome works as an *innate punishment* and is associated with a negative number in the reward function.^4^

In some cases, the value of innate incentives depends on the ongoing motivational state (Toates, 1986). For instance, food is highly desirable when one is food-deprived, but less so after a rich meal. Research has revealed that learning plays a key role in knowing in which motivational states an outcome becomes more or less desirable (Dickinson & Balleine, 1994). In a study on this (Balleine, 1992), food-deprived rats were trained to obtain food after pressing a lever. Later, one group of animals was satiated while another group remained deprived. When exposed again to the lever during extinction (i.e., in a condition where lever pressing did not obtain any food), the two groups pressed the lever an equal number of times. This demonstrates that, even if not hungry, the satiated group still attached a positive value to food, the reason being that it had previously experienced food only in a deprived state. On the contrary, when the two groups were exposed to the lever but now not in extinction (i.e., when they received food after pressing), the satiated group stopped responding after few trials. In this scenario, by experiencing food while not hungry, the satiated group could learn that food was not valuable in this state, realizing that responding was useless.

### Which innate incentives are characteristic of the human species?

Which outcomes are universally appraised as good or bad by people across different cultures? Scholars agree that, by virtue of being critical for satisfying basic biological needs, outcomes such as food, water, sexual partners, and painful stimuli, work as innate incentives virtually for every person (of course, when the organism is in the appropriate motivational state; Toates, 1986). Besides these, psychologists have speculated about other possibilities. An influential proposal is the one made by Maslow (1943, 1954), who argued that humans are driven by a set of innate motives arranged hierarchically, with an individual seeking to fulfil a motive high in the hierarchy only if motives below are already accomplished. In this framework, innate motives include, from bottom to top, physiological needs, safety, love and belonging, self-esteem, cognitive needs such as intellectual stimulation and exploration, aesthetic needs of beauty and order, and self-actualization, that is, the need to achieve one’s talents and interests. This work has inspired other scholars to propose alternative taxonomies (Csikszentmihalyi, 1988; De Charms, 2013; Kenrick et al., 2010), for example an influential one introduced by Deci and Ryan which focuses on competence, relatedness, and autonomy (Deci & Ryan, 2000). Does empirical research lend any support to the existence of innate motives that go beyond basic physiological needs? Available evidence corroborates the existence of at least two of such motives: social motives and, employing Maslow’s terminology, cognitive motives (sometimes also referred to as intrinsic motivations; Ryan & Deci, 2000). Innate social motives are documented by data showing that, in all cultures investigated, very young children spontaneously enact prosocial behavior and seek bonding, joint attention, and collaboration with adults (Padilla-Walker & Carlo, 2015; Tomasello, 2019; van Ijzendoorn & Sagi-Schwartz, 2008). Meanwhile, innate cognitive motives are supported by evidence indicating that very young children are often spontaneously motivated by aspects such as curiosity, novelty, and surprise, even in the absence of any other benefit (Chandler & Connell, 1987; Gottfried, 1983; Liquin & Lombrozo, 2020; Oudeyer & Kaplan, 2009; Ryan & Deci, 2000).

### To what extent are innate incentives stable for each person? And to what extent are they equal across people?

Although innate incentives concern virtually everyone, substantial variability may exist regarding how much value each person assigns to them. For example, assuming that sweet flavor is universally rewarding, it is to be expected that some people are more attracted by it than others. Moreover, although the definition of innate incentives implies that these are inbuilt and universal, experience may nonetheless have a substantial impact. As an extreme example, consider the case of addictive drugs. Insofar as they elicit a euphoric state in virtually all people in all cultures, addictive drugs can be viewed as instances of innate rewards. The impact of experience on the perception of addictive drugs is evident when considering the effects of repeated drug consumption. This elicits neurophysiological changes that affect the value attributed to the drug in a way that produces clinical implications such as tolerance and withdrawal, as for instance described by the incentive-sensitization theory of addiction (Robinson & Berridge, 2025).^5^ Although less dramatic, comparable changes are likely to occur following experience of any innate incentive (as another example, think to the long-term adaptations associated with chronic pain; Baller & Ross, 2017).

While acknowledging the importance of individual differences and experience, we argue that it is nonetheless useful to employ the term *innate* when describing certain rewards and punishments, because this highlights the evolutionary role of such outcomes in providing constrains within which the human species has operated during its evolutionary history. The idea is that, in other words, innate incentives have been established during evolution because of their effect on improving the species’ fitness. Experience and individual differences may modulate the value of these incentives, even dramatically, but they are unlikely to erase such value altogether.

Now that the role of innate incentives has been examined, we move on to analyze learning processes able to transform previously neutral outcomes into *acquired incentives*.

## Conditioned reinforcement

Is the reward function that drives decision-making predicated solely on innate incentives? Or, rather, can we identify learning processes whereby previously neutral outcomes become acquired incentives and, in so doing, inform the reward function during decision-making? We begin exploring this question by examining an empirical phenomenon referred in the literature as *conditioned reinforcement* (Hendry, 1969; Wike, 1966; Williams, 1994a, 1994b). In a standard paradigm investigating it (Wike, 1966), rats initially undergo a classical conditioning procedure where presentation of a neutral stimulus, such as a visual cue, is followed by food. At the end of the session, by eliciting a conditioned response (CR) such as salivation, the visual cue has become a conditioned stimulus (CS). Next, during test, the animals are presented with a lever and, if they press it, they are shown the CS in the absence of food. The result is that rats press the lever repeatedly to obtain the CS. Insofar as it steers an action despite being initially a neutral stimulus, the CS has now become a conditioned reinforcer.

**Fig. 2:**
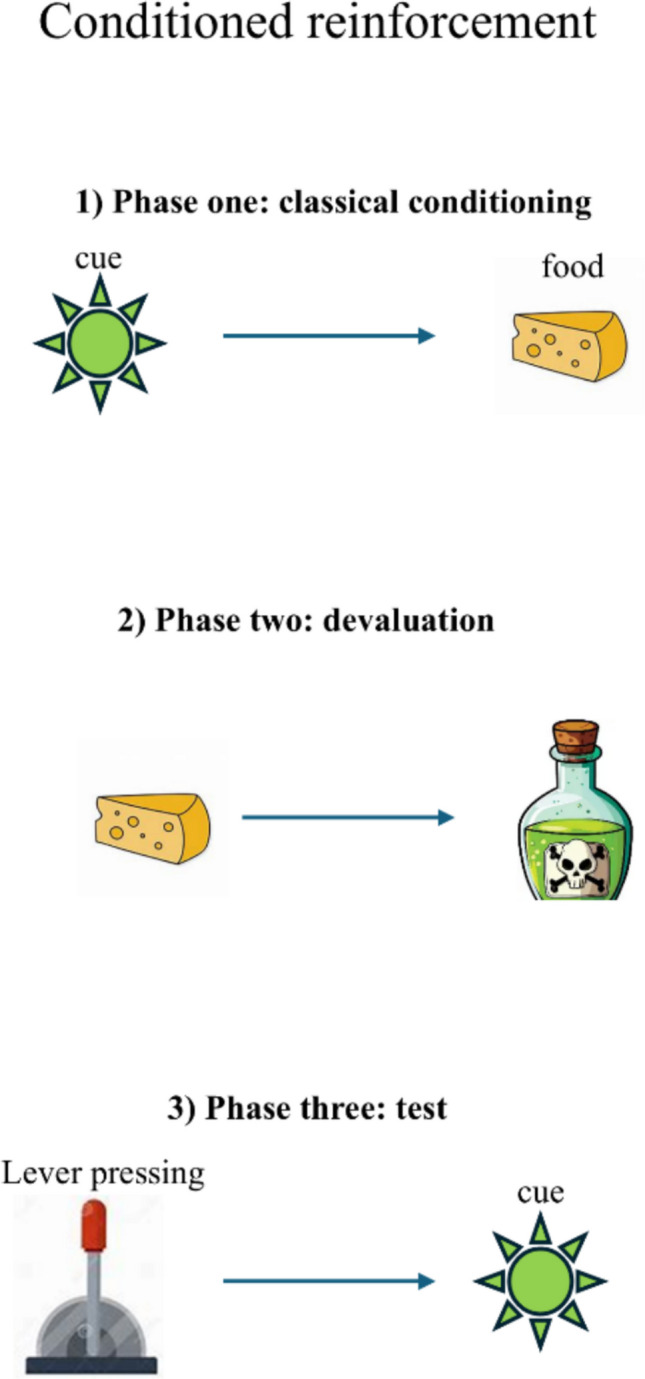
Schematic illustration of the conditioned reinforcement paradigm employed by Parkinson and colleagues (2005). Here, a classical conditioning phase, where food is delivered after presentation of a visual cue, is followed by a devaluation phase, where food is paired with a chemical (lithium chloride) that produces sickness, thus devaluing food. During the test phase, performance of an instrumental action (lever pressing) leads to the presentation of the visual cue employed during the classical conditioning phase. (Color figure online)

At first glance, evidence like the one just described suggests that conditioned reinforcement is a case where *acquired incentives* are learnt and subsequently guide decision-making. Yet, based on the evidence discussed so far, this conclusion appears to be premature: It is possible that, during the classical conditioning phase, animals associate the CS with food, and that, during test, they seek to obtain the CS because they ultimately expect to receive food. Put another way, in the decision tree employed by animals during test, the CS may still be treated as a neutral outcome and as a means to an end. To rule out this possibility, an experiment should attempt to devalue food during the test phase and assess the ensuing impact on conditioned reinforcement (Fig. 2). This experiment has been performed by Parkinson and colleagues (2005) and later supported by K. A. Burke and colleagues (2008). The paradigm employed by Parkinson and colleagues (2005) was similar to the one described above except that, after the classical conditioning phase, animals were administered a chemical (lithium chloride) in conjunction with the food employed during classical conditioning (in this case, sugar dissolved in water). This manipulation is known to link food with sickness, thus devaluing food. According to the hypothesis that the CS steers action because it is associated with food, rats should stop responding during test, given that food was not valuable in this phase. By contrast, if they sought to obtain the CS in and of itself, independent of its relationship with food, animals were predicted to respond nonetheless. This is exactly what happened (Parkinson et al., 2005). This evidence demonstrates that the CS had become valuable as such, and not as a means to get food, proving that conditioned reinforcement can modify the reward function by creating acquired rewards.

### Conditioned reinforcement chain

The study of Parkinson and colleagues (2005) demonstrates that, after being repeatedly paired with an innate reward such as food, a neutral stimulus can become an acquired reward. This raises the following question: What happens if a new neutral stimulus is repeatedly paired with the acquired reward? Will the new neutral stimulus become another acquired reward? Research on second-order conditioning, another phenomenon described by associative learning literature, speaks to this question (Holland & Rescorla, 1975; Rescorla, 1980). In a study on this (Rescorla, 1980), animals were first trained in a classical conditioning session where a visual cue anticipated food, thus becoming a CS (referred to as CS1). Next, the animals underwent a second classical conditioning phase where an auditory tone anticipated CS1. Despite no food being administered here, at the end of the second conditioning session the animals nonetheless exhibited a CR when presented with the tone; thus, the tone had become a CS too (referred to as CS2). In a third session, food was devalued by satiety or by other procedures (e.g., by administration of lithium chloride). How did this impact behavior? The consequence was that animals did not show any CR when exposed to CS1. However, they continued to respond when exposed to CS2. This is remarkable for at least two reasons. First, it suggests that a neutral stimulus (CS2) can become an acquired reward by being paired with another acquired reward (CS1), even in the absence of an innate reward (note, however, that the impact of devaluing food on CS2 has not yet been explored using a conditioned reinforcement paradigm). Second, it suggests that the value of acquired rewards far removed from the original innate reward (i.e., CS2 compared with CS1) is largely independent of the value of the innate reward. Specifically, the value of CS1 is partially independent of food (as demonstrated by the study of Parkinson and colleagues (2005), showing that CS1 retains conditioned reinforcer properties even when food is devalued), but also partially dependent on it (as demonstrated by Rescorla, 1980, showing that presenting CS1 does not elicit the CR)—in other words, CS1 is viewed both as a means to get food and as an incentive as such. By contrast, because CS2 elicits a CR even when food is devalued, the value of CS2 appears to be fully independent of the value of food; in other words, CS2 is viewed as an incentive but not as a means to obtain food.

### Conditioned reinforcement taxonomy

So far, we have examined cases where an acquired reward is established based on being paired with an innate reward. However, in principle, this is only one among a set of cases. A hypothetical taxonomy of acquired incentives can be derived by considering a two-by-two table where columns discriminate rewards from punishments and where rows discriminate presence versus absence of the associated innate incentive during learning (see Table 1; Mackintosh, 1983). Does empirical research support this taxonomy? Above, we have considered the top-left quadrant, where an acquired reward is established by being paired with presence of an innate reward. The top-right quadrant describes cases where an acquired punishment is established by being paired with the presence of an innate punishment (Table 1). The existence of such cases is backed by evidence showing that rats avoid lever pressing when this response leads to presentation of a CS previously associated with electric shock (Killcross et al., 1997). Support comes also from a study demonstrating that, in a gambling task, humans avoid choosing options which lead to a monetary outcome presented in conjunction with a CS previously associated with punishment (Pittig et al., 2014). Of relevance to this domain are also second-order classical conditioning experiments where rats learnt to associate CS1 with electric shock and, next, CS2 with CS1 (Rescorla, 1980; Rizley & Rescorla, 1972). Here, after the CR in response to CS1 was extinguished by presenting repeatedly CS1 without shock, exposure to CS2 still continued to elicit the CR. This observation supports the view that acquired punishments are avoided in and of themselves, and not solely because they predict innate punishments.

**Table 1:** Conditioned reinforcement taxonomy

	Reward	Punishment
Presence	Acquired reward	Acquired punishment
Absence	Acquired punishment	Acquired reward

The bottom-right quadrant refers to cases where an acquired reward is established by being paired with absence of an innate punishment (Table 1). These cases can be investigated by paradigms where a stimulus anticipates absence of an otherwise expected punishment, for example paradigms where a cue (CS +) signals shock delivery while a different cue (CS-) signals shock absence. Here the question is: does the safety signal (CS-) acquire rewarding properties? Supporting this possibility, a recent body of evidence (Fernando et al., 2014a, 2014b) has shown that animals prefer options that lead to the presentation of CS-, in line with the notion that the latter has become an acquired reward (though research has not yet ascertained that the CS- is sought as such, independent of the fact that it predicts absence of an innate punishment).

The last quadrant, on the bottom-left, describes cases where an acquired punishment is established by being paired with absence of an innate reward (Table 1). Following a logic analogous to the previous case, here the reasoning is that absence of an otherwise predicted reward is appraised as being aversive. Research on this scenario is, unfortunately, not as extensive. Nonetheless, a recent study (Bland et al., 2018) has explored pigeons’ choice between (a) one option leading to a stimulus previously associated with food delivery versus (b) one option leading to a stimulus previously associated with food delivery plus a second stimulus previously associated with food absence. The study reports that the animals preferred the first option, in line with the idea that the stimulus previously associated with food absence had acquired aversive properties and was therefore avoided. Although further research is needed, this observation supports the possibility that acquired punishments can be learnt after being paired with absence of innate rewards.

To conclude, all in all, the evidence available appears to be broadly consistent with a four-quadrants taxonomy where acquired incentives are learnt from presence/absence of innate rewards/punishments (Mackintosh, 1983).^6^

### The learning mechanism

A fundamental question concerns the nature of the learning mechanisms driving conditioned reinforcement. As illustrated above, classical conditioning is at the root of conditioned reinforcement. Specifically, the evidence reviewed above demonstrates that, during conditioned reinforcement paradigms, classical conditioning transforms a previously neutral outcome into an acquired incentive, which subsequently enters the reward function during decision-making. More specifically, conditioned reinforcement can be described as a process unfolding over two steps. The first step corresponds to classical conditioning, during which a previously neutral stimulus acquires *cached value* via model-free reinforcement learning. The existence of such model-free learning is compatible with most reinforcement learning models of classical conditioning, including the influential temporal difference algorithm (Dayan & Berridge, 2014; Sutton & Barto, 1999).^7^ The second step involved in conditioned reinforcement occurs when, during decision-making, the cached value attached to the stimulus via classical conditioning enters the reward function. This implies that, during decision-making, the cached value previously acquired during classical conditioning is embedded within the reward function. In support of this idea, consider again the experiment of Parkinson and colleagues (2005), showing that rats chose the CS even when the food associated with the CS during the preceding classical conditioning procedure was devalued via lithium chloride. This finding indicates that, during classical conditioning, a form of model-free learning was engaged, resulting in the CS acquiring cached value. The proof that the CS had acquired cached value is that, in the subsequent decision-making phase, the CS was sought despite the food being devalued. The fact that the CS was sought in the decision-making phase, furthermore, indicates that its cached value was transformed into the reward function adopted during decision-making.

A possible criticism of this interpretation is that cached values cannot enter the reward function because, by definition, the reward function does not deal with cached values. However, this criticism appears to be too strict. In one sense, contemplating the possibility that the reward function can be learnt at all requires being open to the possibility that cached values (which indicate how stimuli acquire value as such, as not by virtue of their link with other stimuli) can enter the reward function. Indeed, if this view is accepted as a possibility, then the evidence on conditioned reinforcement overviewed here fully supports this possibility—specifically, it supports the notion that, during decision-making, cached values acquired during classical conditioning are later translated into the reward function.^8^

One last point is worth discussing here. Consider again the finding that conditioned reinforcement can occur also when the innate reward used during classical conditioning (e.g., food) has been devalued (Parkinson et al., 2005). A key question is in which conditions conditioned reinforcement is resistant to devaluation as in the case documented by Parkinson and colleagues (2005). With this regard, research has revealed that instrumental behavior is resistant to devaluation when training is long but not when training is short (e.g., Balleine & O’Doherty, 2010; Daw et al., 2011). A similar pattern may emerge also for conditioned reinforcement, a possibility that nevertheless remains to be explored empirically.

### Formal models

Once it is established that classical conditioning can lead to the formation of acquired incentives which, during decision-making, inform the reward function, the next question is how this process works in detail. Insight on this is offered by mathematical models of classical conditioning which examine how CSs garner cached value. This literature is vast and encompasses various disciplines including associative learning and reinforcement learning (Courville et al., 2006; Gershman & Niv, 2012; Pearce & Hall, 1980; Rescorla & Wagner, 1972; Sutton & Barto, 1999). Though a detailed overview of this is beyond the scope of the manuscript, it is helpful to report at least one of the most common mathematical formulations employed (Rescorla & Wagner, 1972):

$$V\left(t+1\right)=V\left(t\right)+\alpha \left(R\left(t\right)-V\left(t\right)\right).$$

This, referred to as the Rescorla–Wagner rule, posits that the value of the CS on the next trial (V(t + 1)) is equal to the current value of the CS, V(t), plus a learning rate (the parameter α) multiplied by the prediction error (i.e., by the difference between the innate incentive, R(t), and the current value of the CS, V(t)). Though this formulation is not without shortcomings (Courville et al., 2006; Gershman & Niv, 2012; Pearce & Hall, 1980), it can account for various empirical observations including the fact that a CS is appraised as more valuable when the associated innate incentive has higher value and probability and when it is presented sooner (the latter aspect can be captured simply by adding a delay discounting parameter to the equation; Rescorla & Wagner, 1972).

This and similar models of classical conditioning are of obvious significance for the topic explored here, yet two potential problems should be highlighted. First, these models have rarely been assessed in conditioned reinforcement paradigms. Second, as highlighted by Dayan and Berridge (2014), most of these models do not distinguish between model-free and model-based components of classical conditioning. As illustrated above, the evidence indicates that, after classical conditioning, CSs acquire cached value independent of the innate incentives they anticipate – as documented, for example, by Parkinson and colleagues (2005). This requires a form of model-free learning, which is the aspect captured by most formal models of classical conditioning such as the Rescorla–Wagner rule. However, evidence also suggests that CSs are not only valuable as such, but also to the extent that they predict their associated innate incentives – as documented, for instance, by Rescorla (1980). This aspect requires a form of model-based learning based on storing stimulus–stimulus associations. Most mathematical models of classical conditioning have neglected the model-based component (Dayan & Berridge, 2014). Yet to fully understand how classical conditioning contributes to the formation of acquired incentives during decision-making, it is important to develop computational models that tease apart model-free and model-based learning processes at play during classical conditioning.

### Special cases of conditioned reinforcement

Skinner (1953), an eminent behaviorist psychologist, conjectured that, if a stimulus is paired with multiple types of innate rewards (e.g., water and food), then during subsequent decision-making the stimulus becomes a more valuable acquired reward compared with a stimulus previously paired with one type of innate reward only (e.g., food).^9^ According to this view, examples of stimuli paired with multiple types of innate rewards (referred to as *generalized reinforcers*) are abstract social cues such as money, sentences like “This is very good,” and high grades at school (Skinner, 1953; Wike, 1966). To date, though, experimental testing of Skinner’s hypothesis has provided at best modest support, and a definitive proof that generalized reinforcers have special properties is still missing (Wike, 1966).

As an alternative to Skinner’s proposal, a recent model (Rigoli, 2022) has conjectured that a stimulus becomes a more valuable acquired reward when it predicts an innate reward in multiple contexts, compared with a stimulus predicting an innate reward in one context only. For example, according to this hypothesis, money will be more valuable for someone who uses them to buy goods in different town markets, compared with someone using them to buy goods in one market only. Although this possibility fits with circumstantial evidence (Rigoli, 2022), a systematic empirical testing of it remains to be carried out.

The notion that a stimulus becomes a more valuable acquired reward when it predicts an innate reward in multiple contexts implies the existence of generalization processes, namely, of processes able to infer which acquired incentives apply to multiple contexts. Such generalization processes may shed light on another important question—that is, the question of whether abstract concepts can become acquired incentives via conditioned reinforcement. Take abstract concepts such as “being generous,” “respecting tradition,” or “being humble.” Assuming that these concepts do not have any innate value a priori, can they become ends in and of themselves via conditioned reinforcement? The answer to this question is not straightforward, given that, both theoretically and empirically, the construct of conditioned reinforcement has been applied to concrete stimuli like visual or auditory cues. Some abstraction process seems to be required for abstract concepts to work as acquired incentives, but it is not clear how this process might work. A possibility is that the human brain is capable to develop abstract incentives by generalizing over concrete incentives. To illustrate, consider a person who, thanks to conditioned reinforcement (supported by innate reward predicated in terms of social approval), has learnt to appreciate attending religious services, wearing traditional clothes, and behaving submissively towards their parents. The person’s brain, according to this view, may at some point activate a generalization process that seeks to infer which abstract incentive underlies the more concrete incentives. The result of this inference may be the conclusion that “respecting tradition” is the underlying abstract incentive. In turn, this may spur the person to pursue this more general incentive thereafter, even when exposed to different situations. This is an explanation of how abstract incentives (respecting tradition) may emerge based on more concrete incentives (attending religious services, wearing traditional clothes, being submissive towards one’s parents). Yet it is important to stress that this explanation is at present speculative and remains to be tested empirically. Research on how generalization processes shape the emergence of abstract incentives should consider contemporary models of generalization in cognitive sciences (Wu et al., 2024), especially those developed in the context of decision-making (Schulz et al., 2018; Wimmer et al., 2012; Wu et al., 2018).

### Extinction

An important question is how persistent the value attributed to an acquired incentive is. Speaking to this, research on conditioned reinforcement has found that this phenomenon is subject to extinction, since an acquired incentive progressively ceases to motivate choice behavior after being repeatedly experienced without any innate incentive (Wike, 1966). Does extinction imply that the acquired incentive has lost its value? Animal research suggests that this may not necessarily be the case. A remarkable phenomenon highlighted by the literature on classical conditioning is reinstatement occurring after extinction. Reinstatement emerges when, even if a CR in response to a CS has been extinguished, the CR reappears again when the CS is presented in a different context (e.g., in a different experimental chamber; Bouton, 2004; Bouton et al., 2011). This suggests that, although the CR is extinguished in one context, the CS remains valuable and capable to motivate behavior in other contexts. Something analogous may occur for acquired incentives: even if they stop motivating choice behavior in one context, they may still retain their value and operate in different contexts. However, whether acquired incentives are subject to reinstatement and analogous phenomena remains an open empirical question.

Research on classical conditioning has reported that, after being extinguished, a CR can sometimes resurface, so to speak, spontaneously, even without any apparent change in the environment (Bouton, 2004; Bouton et al., 2011). Some scholars have interpreted this spontaneous reappearance as due to a change in the temporal context, even when the spatial context has remained unaltered (Bouton, 2004; Bouton et al., 2011). This phenomenon can explain why, for instance, maladaptive fear responses are sometimes hard to inhibit even if no danger has been experienced for a while (Glotzbach-Schoon et al., 2015). A similar mechanism may characterize conditioned reinforcement, since acquired incentives may simply resurface after some time simply because the temporal context has changed. Again, however, we stress that this hypothesis is speculative since the nature of extinction in conditioned reinforcement remains to be studied systematically.

Further insight regarding the persistence of acquired incentives comes from research on human ethical values. A large body of evidence suggests that humans acquire their ethical values in infancy and adolescence, and, to a large extent, stick with them in adulthood (Bardi & Goodwin, 2011; Block & Block, 2006; Cieciuch et al., 2016; Fraley et al., 2012; Inglehart, 2018). This hints to the possibility that acquired incentives may largely arise early in life and be resistant to change thereafter. In other words, both acquisition of new incentives and extinction of old ones may be less common (though potentially not impossible, especially if radical life changes occur; Lönnqvist et al., 2011) in older age.

### Neurobiology

Neuroscientists have explored the neural substrates underlying conditioned reinforcement. They have found that the brain regions that control conditioned reinforcement correspond to the same regions involved in various aspects of reward processing, including the orbitofrontal cortex, the amygdala, and the dopaminergic projections from the ventral tegmental area to the nucleus accumbens.

Pears and colleagues (2003) have reported that, after lesioning the orbitofrontal cortex, rats fail to learn an instrumental response that leads to a CS while being able to learn an instrumental response that leads to an innate reward (see Cox et al., 2005, for a related finding in humans). This finding highlights the involvement of the orbitofrontal cortex in supporting conditioned reinforcement. Meanwhile, various studies in rodents (Burns et al., 1993; Cador et al., 1989; Gewirtz & Davis, 1997; Meil & See, 1997; Whitelaw et al., 1996) and monkeys (Parkinson et al., 2001) have reported that the amygdala, and specifically its basolateral portion (comprising the lateral, basal, and accessory basal nuclei), is implicated in conditioned reinforcement as well. Finally, a large body of evidence shows that dopaminergic signaling from the ventral tegmental area to the nucleus accumbens is also important in this domain. It has been found that, when addictive drugs boost the dopaminergic activity in the nucleus accumbens, conditioned reinforcement is enhanced (Parkinson et al., 1999; Taylor & Robbins, 1984; Wolterink et al., 1993). Meanwhile, research has observed that manipulations of acetylcholine receptors and NMDA receptors in the ventral tegmental area alter conditioned reinforcement (Löf et al., 2007, 2010; Wickham et al., 2015).

Although the neuroscientific literature overviewed so far is very relevant, it does not address a critical aspect analyzed in the paper, namely, the issue concerning whether, during conditioned reinforcement, a CS is sought as an end in and of itself (as documented by Parkinson et al., 2005) or as a means to obtain an innate reward. So far, only Burke and colleagues (2008) have examined this issue explicitly, with a focus on the orbitofrontal cortex. Adopting a complex experimental paradigm where the rats’ orbitofrontal cortex was lesioned, these authors have revealed that this brain region encodes the association between the CS and the innate reward but does not represent the value of the CS in and of itself. In other words, these findings demonstrate that the orbitofrontal cortex contributes to conditioned reinforcement when the CS is viewed as a means to achieve an innate reward, but not when the CS is sought as an end. This suggests that the orbitofrontal cortex is not implicated in representing acquired incentives. Unfortunately, at present similar studies are unavailable regarding the other brain regions involved in conditioned reinforcement. Some scholars have speculated that the basolateral amygdala is critical in the acquisition of cached values linked with CSs and in treating these CSs as valuable as such during decision-making (Burke et al., 2008; Pears et al., 2003). However, this hypothesis remains to be tested empirically. A possibility is that dopaminergic signaling from the ventral tegmental area to the nucleus accumbens may also play a role. With this regard, available research indicates that dopamine is critical both for model-free learning engaged during classical conditioning (e.g., Schultz et al., 1997), which may drive the acquisition of acquired incentives driving conditioned reinforcement, as well as in model-based learning (e.g., Gardner et al., 2018; Sharpe et al., 2017), which instead may be implicated in learning the association between the CS and the innate reward (e.g., by storing the sensory properties of the innate reward; Fry et al., 2025; Holland, 1990). As for the amygdala, however, the contribution of dopamine in encoding acquired incentives during conditioned reinforcement remains to be investigated empirically.

### Conditioned reinforcement and evolution

One last fundamental question is what evolutionary advantage, if any, is afforded by conditioned reinforcement. It is reasonable to assume that, in evolutionary terms, for humans it is advantageous to approach innate rewards (e.g., food) and to avoid innate punishments (e.g., pain). But what is the advantage of approaching acquired rewards and of avoiding acquired punishments? Why do humans not simply care about innate incentives and treat acquired incentives as means? After all, in an evolutionary perspective, this is what they really are. Yet the evidence reviewed so far convincingly demonstrates that acquired incentives are treated as ends by humans, as they are appraised as valuable as such. Why? A possible answer to this question may be linked with the limited capacity of the brain combined with the complexity of typical human tasks (Daw et al., 2005). The classical example is the game of chess: planning the next move by combining all the different possibilities requires a gigantic decision tree which is computationally intractable—hunting was probably no less complex for our hunter-gatherer ancestors. The brain must find a way to simplify the problem, and conditioned reinforcement may offer a strategy to do so. Realizing that a neutral stimulus (e.g., money) is, in most contexts, conducive of innate rewards might offer a viable computational shortcut: ignoring the context and just seeking the stimulus can simplify the problem substantially. A similar logic is employed by algorithms in computer science and in reinforcement learning where a complex task is broken down into a sequence of subgoals and where planning is aimed at reaching the ongoing subgoal, rather than the final goal, reducing greatly the computational demand (Botvinick et al., 2009; Newell & Simon, 1972; Solway et al., 2014). Conditioned reinforcement might have evolved as a similar strategy: Acquired incentives may be functionally akin to subgoals and thus greatly reduce the problem complexity.^10^ This could have enabled the brain to employ decision-making strategies which are computationally tractable and yet effective.

In conclusion, this section has examined how conditioned reinforcement contributes to shape the reward function by driving the formation of acquired incentives. The next section proceeds by considering another key process that may be at play in this domain, the process of social learning.

## Social learning

A vast literature has shown that social learning plays a much greater role in humans compared with other animals, leading some scholars to define human beings as ultra-social animals (Tomasello, 2014, 2019). The evidence indicates that an individual’s knowledge of the physical and cultural environment, as much as of the skills necessary to navigate it, is, to a large extent, the result of interactions with other people (e.g., Boyd et al., 2011; Tomasello et al., 2005). In the terminology employed in the introduction to describe decision-making, social learning plays a pivotal role in the formation of the transition function characterizing one’s decision trees. Yet less is known about whether social learning plays a similar role also in shaping the reward function. In other words, to what extent does social learning contribute to shape the reward function? More specifically, to what extent does other people’s behavior contribute to the formation of acquired incentives? To address these questions, this section explores scenarios where other people’s actions may contribute to shape the acquisition of incentives (Olsson et al., 2018).

The first scenario occurs when other people deliver rewards or punishments in response to one’s actions. For example, parents may employ a cake to reward children who succeed at an exam, while punishing them by withholding the cake if they fail the exam. The processes underlying this scenario are arguably the same as those examined above when discussing the notion of conditioned reinforcement (Matyjek et al., 2020). For example, after some experience of the cake following exam success, the latter might become an acquired reward for children and be appraised as valuable in and of itself.

### Vicarious learning

Not only other people can shape acquisition of incentives by rewarding or punishing an agent, but also simply by displaying their behavior, even if the agent is not directly rewarded nor punished. Put another way, observing other people’s actions appears to influence an agent’s subsequent behavior—a phenomenon referred to as *observational learning* (Bandura, 1977; Fryling et al., 2011; Greer et al., 2006). Different forms of such learning have been documented empirically. A first form, described as *vicarious learning* (Bandura, 1977; Morelli et al., 2018), occurs when an agent observes a model (i.e., another human being) whose actions are rewarded or punished. Typically, in this scenario the agent later exhibits the rewarded model’s actions and inhibits the punished model’s actions. Conducted by Bandura and colleagues (1963), a famous study on vicarious learning began by showing children a video of a model performing aggressive actions against a doll. For one group of children, the model was next rewarded, whereas for another group of children the model’s behavior was punished. In a test phase, compared with a control group including children who watched no video, children of the reward group were more likely to express aggressive behavior against the doll. By contrast, compared with the same control group, children of the punishment group were less likely to express aggressive behavior against the doll. These and similar findings (Bandura, 1977) demonstrate that, even if not directly rewarded or punished, people can learn an action repertoire simply by watching others’ behavior being rewarded or punished.

Let us assess vicarious learning by asking the following question: which specific information is learnt during this process? More specifically, does an agent acquire any new incentive during vicarious learning? The answer to this question may be negative. The reason is that, during vicarious learning experiments, an agent is exposed to a model who receives incentives which are already established (i.e., innate incentives or acquired incentives which have been developed beforehand). Therefore, the most parsimonious interpretation is that, by observing the model, an agent learns nothing but that a certain action leads to a well-established incentive. In other words, the person arguably acquires knowledge about the means to get to an end, while learning nothing about the ends themselves. Employing the terminology used in the introduction, during vicarious learning the person updates the transition function, not the reward function.

Yet although most research on vicarious learning does not speak to the question of how the reward function is learnt, a small number of studies does. These are studies that have documented the existence of conditioned reinforcement occurring vicariously (Arenson, 1976; Leaf et al., 2012; McGinley & Murray, 1979). In one of these studies (Arenson, 1976), children were divided into three groups: one (*direct reward group*) which, after performance of an appropriate action, was presented with a visual cue followed by reward; one (*observed reward group*) which observed other children who, after performance of an appropriate action, were presented with a visual cue followed by reward; and a control group including children who were not exposed to any cue nor to reward. A test phase revealed that, in comparison with the control group, both the direct reward group and the observed reward group were more likely to perform an action leading to the visual cue, even in the absence of reward—supporting the idea that the cue had become a conditioned reinforcer also for the observed reward group. However, does this demonstrate that children from the observed reward group valued the cue in and of itself? In other words, does this demonstrate that the cue had become an acquired reward? Or, rather, was the cue sought because of its association with the initial reward? As discussed above, addressing this question requires to assess whether the conditioned reinforcer is still desired after the associated innate reward has been devalued. Unfortunately, this procedure has not been applied in this domain, implying that whether acquired incentives can be established via vicarious conditioned reinforcement remains to be demonstrated conclusively.

### Spontaneous imitation

Strikingly, observational learning appears to occur also in scenarios where the model is not rewarded nor punished (Bandura, 1977; Fryling et al., 2011; Greer et al., 2006). Once again, the pioneering work of Bandura et al. (1961) offers some of the earliest evidence on this phenomenon. In an experiment similar to the one described above (Bandura et al., 1961), one group of children observed a model performing aggressive actions against a doll, but now without the model being rewarded nor punished for doing so. The test phase showed that, in comparison to a control group of children who had watched no model, the participants exposed to the model were later more likely to exhibit aggression against the doll. These and similar findings (Bandura, 1977) demonstrate a human inclination to imitate observed actions, even when the model initially performing these actions was not rewarded – we can call this phenomenon *spontaneous imitation*. This raises the following question: what are the processes motivating spontaneous imitation? And, more to the point, does acquisition of new incentives drive spontaneous imitation?

To answer this question, let us assess the two major theories concerning the motivational processes that drive spontaneous imitation. According to the first one, spontaneous imitation is controlled by a specific neural mechanism (often associated with the mirror neurons system) which predisposes an agent to automatically copy the movements performed by an observed conspecific (Heyes & Ray, 2000; Iacoboni, 2009; Meltzoff & Moore, 1997; Rizzolatti et al., 2001). Thought to facilitate joint action and cooperation, such automatic imitation system is often described as controlled by stimulus-action associations, not by goal-directed behavior. This explanation works well when describing forms of spontaneous imitation occurring online and automatically, such as when one’s facial expression and posture are tuned to those of an interlocutor. According to this view, spontaneous imitation does not involve acquisition of new incentives since it does not even recruit goal-directed processes.

The second theory about the motives underlying spontaneous imitation stresses the central role played by social motivations (Nielsen et al., 2008; Over & Carpenter, 2012; Tomasello et al., 2005). The proposal is that humans employ imitation as a means to achieve innate social rewards such as bonding with the observed model, signaling group membership, or displaying action mastery. During imitation, people may extend their action repertoire and acquire new skills, but, according to this view, the incentives driving spontaneous imitation remain nonetheless innate social rewards. This explanation fits well with circumstances where imitation has a clear function in regulating social relationships, such as during play, rituals, or during athletic competitions (Nielsen et al., 2008; Over, 2016; Over & Carpenter, 2012; Tomasello et al., 2005). According to this theory, spontaneous imitation does not involve acquisition of new incentives because innate social rewards are the only motivational factors at play. For example, after observing a friend playing a new game, a child may start playing the same game not because the child is attracted by the game as such, but because the child seeks to bond with the friend—an instance of an innate social reward.

Do the two theories just described account for all cases of spontaneous imitation? If this is true, then acquisition of new incentives plays no role in this phenomenon. A careful look, however, suggests that some cases may fall outside the remit of the two theories. These cases have two joint characteristics. First, and contrary to the first theory above, in these cases spontaneous imitation is not due to stimulus-action associations, but to goal-directed behavior. The existence of such cases has been documented by a large body of evidence showing that, rather than simply reproducing the model’s movements, imitation is sometimes flexible enough to be tuned to achieving the ends inferred from the model’s behavior (Tomasello et al., 2005).^11^ Second, and contrary to the second theory above, in these cases imitation arises in the absence of obvious innate social rewards (Fridland & Moore, 2015), for example when a person is alone – think to the aforementioned experiment of Bandura, where children imitated aggressive behavior later when placed in a non-social environment (Bandura et al., 1961). The two theories, therefore, struggle to explain cases of spontaneous imitation occurring in non-social contexts and tuned to obtain the model’s inferred ends.

**Fig. 3:**
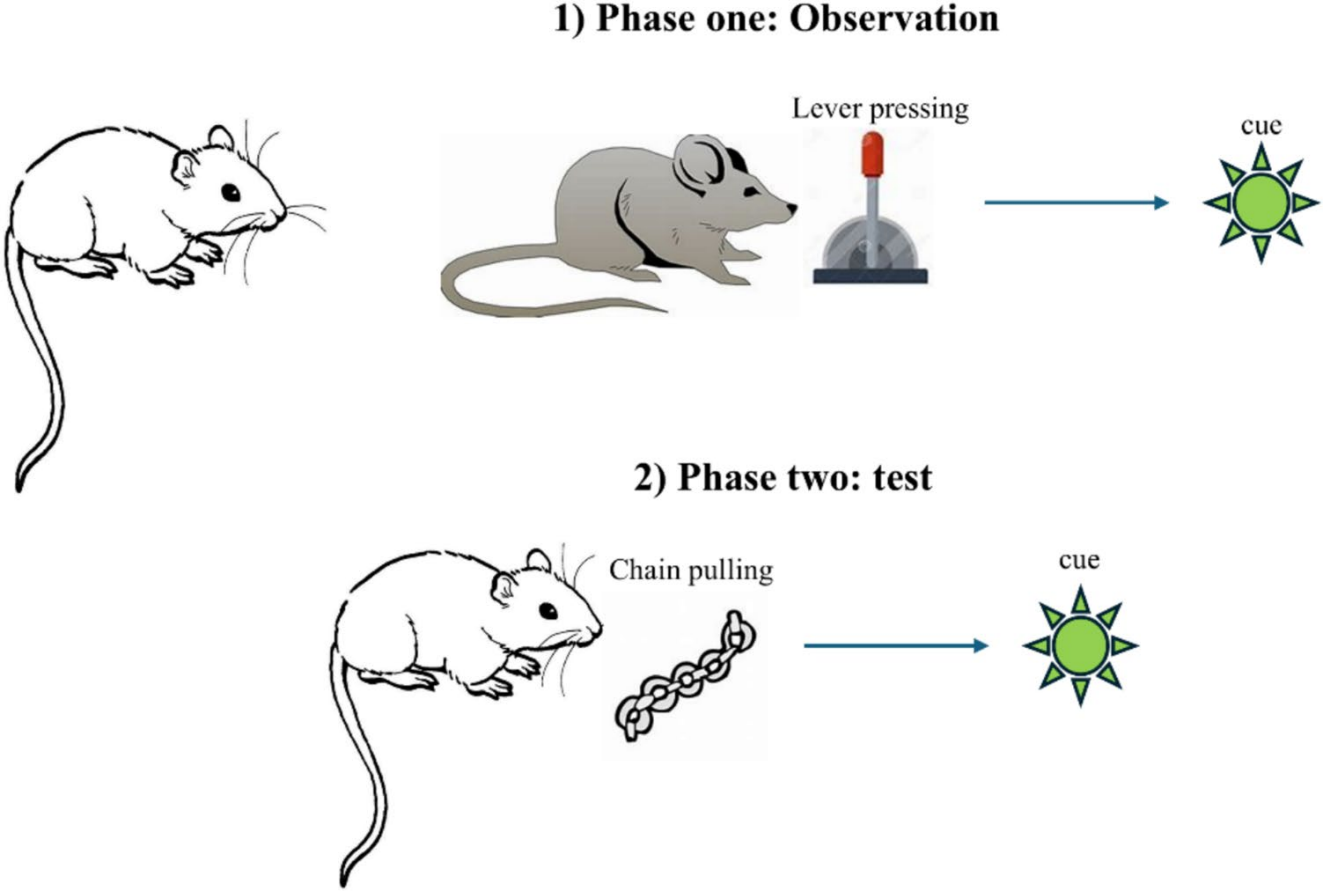
Schematic illustration of imitative incentive learning. Here, during the first phase, an agent observes a model who performs an instrumental action (lever pressing) to obtain a visual cue. Next, during the test phase, the animal is given the opportunity to perform a different instrumental action (chain pulling) to obtain the visual cue presented during the observation phase. (Color figure online)

How can we explain such cases? We suggest the following possibility: after inferring the ends sought by other people from observing their behavior, humans may be predisposed to acquire such ends for themselves – these, in other words, would be transformed into new incentives. Put simply, humans may be predisposed to appropriate the incentives pursued by other people. In the example of the aforementioned experiment of Bandura and colleagues (1961), by observing the model attacking the doll, children may infer that damage to the doll was viewed as an incentive by the model, and they may appropriate that incentive for themselves. Next, when given the opportunity, they may pursue the same incentive, thus manifesting aggressive behavior during the test phase. Note that, in contrast with other explanations, the one proposed here implies that newly acquired incentives underpin spontaneous imitation. Remarkably, this highlights a form of learning which is different from conditioned reinforcement. The latter entails that a new incentive is acquired by being, directly or vicariously, paired with an innate incentive during learning. Here, by contrast, a new incentive is acquired by observing other people pursuing an incentive—in other words, here there is no pairing of a neutral stimulus with an innate incentive during learning. We can refer to this new form of learning as *imitative incentive learning* (Fig. 3). The concept of imitative incentive learning is akin to recent ideas proposed by Ho and colleagues (2017) and by Wu and colleagues (2022) who have conjectured that observing other people’s actions may influence value. These previous proposals, however, have considered the notion of value in general, and not within the domain of decision-making in particular. Therefore, they have not assessed the specific question of whether observing other people’s actions affects the acquisition of new incentives within the reward function. By contrast, the concept of imitative incentive learning focuses specifically on how the reward function is acquired and used during decision-making—this specific focus reflects the novelty of this construct in the context of prior literature.

### Imitative incentive learning

Is there any unambiguous empirical evidence documenting the existence of imitative incentive learning? A first body of work relevant to this question comes from social psychological investigations on the so-called goal contagion phenomenon (Aarts et al., 2004; Brohmer et al., 2021; Nook et al., 2016). In a study on this (Aarts et al., 2004), a screening procedure allowed researchers to divide participants into two groups, one including people who, in general, are interested in money (money group), the second including those who are not interested in money (control group). Both groups were exposed to two vignettes, one describing a person seeking to gain money (money vignette), the other describing something else (control vignette). The authors observed that, in a subsequent task, people from the money group pursued money more after exposure to the money vignette versus exposure to the control vignette; by contrast, no difference emerged for the control group. The interpretation of this finding is that, for people who value money greatly, observing a person seeking money triggers the goal of seeking money. This is an instance of goal contagion, occurring when observing someone pursuing a goal encourages a person to pursue the same goal. Although a recent metanalysis has argued that the evidence on this phenomenon is not yet conclusive, goal contagion has been documented by various investigations (Brohmer et al., 2021). Is the notion of goal contagion compatible with the notion of imitative incentive learning? Despite the apparent similarity, the two constructs are substantially different. Goal contagion concerns the activation of a goal, which presumes that an outcome (e.g., money) is already viewed as an incentive in the first place. Goal contagion prescribes that observing another individual who is pursuing an outcome spurs the agent to do the same, without necessarily altering the value attributed to the outcome. In support of this interpretation, in the study above the effect emerged only for people who already valued money to begin with. By contrast, imitative incentive learning occurs when an outcome is not valuable initially, but becomes valuable after observing someone who pursues it. If anything, therefore, the study above (Aarts et al., 2004) is at odds with the existence of imitative incentive learning, because in that study the effect did not emerge for people who were not interested in money at the beginning. In conclusion, research on goal contagion appears to be inadequate to establish whether imitative incentive learning exists or not.

Another relevant body of work encompasses studies investigating whether an actor’s choices change after the actor has made decisions on behalf of another person (FeldmanHall et al., 2018; Garvert et al., 2015). In one of these studies (Garvert et al., 2015), participants initially played a decision-making task based on temporal discounting (i.e., where participants had to decide between larger rewards collected later and smaller rewards collected sooner). In a second phase, participants played the same task, but now on behalf of a confederate while receiving feedback about the confederate’s preferences on every trial. When, in a third phase, participants played the task once more but now for themselves as in phase one, their preferences had shifted towards the confederate’s preferences as observed in phase two. Altogether, this and similar studies (FeldmanHall et al., 2018; Garvert et al., 2015) reveal that a person’s choices conform to a confederate’s preferences after the person has made decisions on behalf of the confederate while receiving feedback from the confederate. This finding is compatible with imitative incentive learning. Indeed, when making choices on behalf of the confederate, participants receive information about the confederate’s incentives, and, in line with imitative incentive learning, they may acquire those incentives for themselves. Nevertheless, the confederate’s feedback provided in phase two may recruit a different mechanism: when the confederate signals approval (disapproval) for the participant’s choices on behalf of the confederate, this may reward (punish) the participant’s initial strategy, thus changing the participant’s preferences thereafter. If this interpretation is correct, then conditioned reinforcement, and not imitative incentive learning, is the process at play. Since this interpretation cannot be ruled out, this literature does not provide conclusive evidence on the existence of imitative incentive learning. To avoid potential confounds, an ideal paradigm seeking to isolate imitative incentive learning should include no action performed by the actor (and thus no reward nor punishment to the action) during observation of the other person’s behavior.

An attempt in this direction has been made by the recent paper of Najar and colleagues (2020) where participants performed repeated choices between two visual cues, each leading to a monetary gain with a specific probability (for investigations employing a similar approach, see C. J. Burke et al., 2010; Charpentier et al., 2020, 2024; Collette et al., 2017; Jern et al., 2017; Suzuki et al., 2012). In some trials, participants observed a confederate who made a choice between the same two cues. Crucially, and different from the studies mentioned above (FeldmanHall et al., 2018; Garvert et al., 2015), here, no feedback was provided during observational trials. The analyses revealed that participants were influenced by observing the confederate’s choice in a way that is consistent with imitative incentive learning—that is, with the possibility that a cue became more valuable after observing the confederate choosing the cue. Still, an alternative interpretation of these findings cannot be ruled out. The alternative interpretation asserts that, rather than boosting the value of the cue in and of itself, observing the confederate choosing the cue promoted the belief that the cue was conducive of money. In other words, it is possible that the observational trials elicited learning about the *means* to get money, rather than learning about the *value* of the cue. This alternative interpretation cannot be ruled out, since the association between the cues and money was always explicit in the task used by Najar and colleagues (2020).^12^

The literature on observational learning in children has developed a paradigm where this confound does not apply (Greer & Singer-Dudek, 2008; Greer et al., 2008; Singer-Dudek et al., 2011). In one study (Greer & Singer-Dudek, 2008), 3-to-5-year-old children first performed a task where a correct response was followed by reception of a plastic disc. At this stage, receiving the disc did not improve the children’s performance, indicating that the disc was initially appraised as neutral. Next, the children observed peers who, while performing a task, received the plastic disc (the children could not observe the peers’ behavior, but they observed delivery of the disc). In a test phase, the children were asked to perform another task, where, once again, they received the disc following a correct response. The analyses revealed that, now, reception of the disc improved performance. These findings can be explained as manifestations of imitative incentive learning. Indeed, a possibility is that, during the observation phase, children inferred that the peers’ incentive was to obtain the disc. In turn, this might have elicited imitative incentive learning, implying that now children aimed at obtaining the disc too, explaining why at the end their performance improved when it was rewarded with the disc. Two aspects of this study are particularly noteworthy. First, and contrary to Garvert and colleagues (2015) and FeldmanHall and colleagues (2018), in this study the observation phase did not involve any action performed by the observer, ruling out the possibility that conditioned reinforcement may be involved in one way or another. Second, and contrary to Najar and colleagues (2020), participants never experienced any association between the disc and other innate or acquired incentives. Therefore, it can be concluded that the disc was pursued because it had become an end in and of itself, and not because it was viewed as means to obtain other incentives. This and similar studies (Greer & Singer-Dudek, 2008; Greer et al., 2008; Ogulmus et al., 2024; Singer-Dudek et al., 2011) provide the most compelling evidence supporting the existence of imitative incentive learning to date.

In conclusion of our analysis of imitative incentive learning, we speculate on the evolutionary function of this process. A vast body of research on cultural evolution has emphasized the importance of social learning in ensuring that cooperation within the members of society is optimized (Boyd et al., 2011; Henrich, 2016). The same argument can be extended to the case of imitative incentive learning. By appropriating each other’s incentives, people may eventually conclude that it is useful to coordinate their actions in order to pursue their shared incentives.

To summarize, this section has examined how acquisition of new incentives can be influenced by other people. Besides delivering rewards or punishments directly, other people can shape one’s incentives indirectly, via observational learning. In one form thereof referred to as vicarious learning, the observed model is punished or rewarded after performing an action. Empirical evidence indicates that, during vicarious learning, new incentives can be acquired in a way akin to conditioned reinforcement. Another form of observational learning occurs when, although the observed model is not rewarded nor punished, the model’s behavior is nonetheless imitated—a phenomenon labelled as spontaneous imitation. We have pointed to empirical evidence suggesting that sometimes spontaneous imitation might be mediated by acquisition of new incentives. Notably, these new incentives might be learnt via a process different from conditioned reinforcement—that is, via imitative incentive learning, whereby the observed model’s incentives are appropriated for oneself.

## Discussion

We have concluded our analysis concerning the learning processes that shape the reward function. The available evidence indicates that the reward function not only includes innate incentives but also acquired incentives—that is, outcomes that are treated as ends despite being neutral at the outset. Our investigation reveals the existence of two basic forms of learning underlying the emergence of acquired incentives. The first is conditioned reinforcement, occurring when a neutral stimulus is paired with an innate incentive (or possibly with another acquired incentive). The consequence of such pairing is that the neutral stimulus becomes an acquired incentive in subsequent decision-making. Conditioned reinforcement not only occurs with direct experience but it can also occur vicariously—that is, by observing another person exposed to a neutral stimulus anticipating an innate incentive. Observation is also at the root of the second form of learning: imitative incentive learning. This occurs when, after an agent has inferred the incentive pursued by another person, the agent appropriates the incentive for oneself.

An important question is whether conditioned reinforcement and imitative incentive learning cover the full gamut of learning processes that can shape the reward function. A potential additional candidate is learning via verbal instruction. There is abundant evidence showing that people often pursue certain outcomes because they have been instructed to do so. Yet whether verbal instructions recruit learning mechanisms different from conditioned reinforcement or from imitative incentive learning remains dubious. Consider a person who has been instructed to pursue an outcome (e.g., a visual cue) which, initially, was neutral. Will the person start seeking the outcome as such after receiving the instruction? Empirical research has yet to demonstrate that this is the case. At present, a possibility that cannot be discarded is that, after receiving instructions, people pursue certain outcomes as means to collect innate rewards (e.g., social approval). Even if research eventually demonstrates that an outcome can become an acquired incentive after instructions, it remains questionable whether the learning processes involved are different from conditioned reinforcement or from imitative incentive learning. Indeed, conditioned reinforcement may be engaged: verbal instructions may establish an association between the outcome (e.g., a visual cue) and an innate reward (social approval), which in turn may transform the outcome into an acquired incentive. Or instructions may signal that another individual (the source of instructions) desires the outcome, thereby recruiting imitative incentive learning—likewise, transforming the outcome into an acquired incentive. Based on this line of reasoning, we conclude that it is doubtful that verbal instructions enroll learning processes that differ from conditioned reinforcement or from imitative incentive learning. Still, it should not be excluded that, besides conditioned reinforcement and imitative incentive, other learning processes contribute to shape the reward function. As stated above, the question of how the reward function is formed has rarely been studied, and therefore various aspects remain to be elucidated. An interesting research avenue is to examine the possibility that additional forms of learning are involved in this domain.

Relatedly, an important question is how useful conditioned reinforcement and imitative incentive learning are in explaining decision-making in everyday life. When facing concrete everyday life scenarios (e.g., when trying to explain why John wants to start playing basketball), it is probably impossible to know for sure whether an end has emerged because of conditioned reinforcement (in the past John has been praised for playing basketball), imitative incentive learning (John’s friends all want to play basketball), or because of other unknown forms of incentive learning. Moreover, the processes at play in ecological settings may interact in complex ways. Take the case of a child observing other kids doing math exercises at school. Witnessing a peer being punished for stopping doing the exercises may motivate the child to engage with the exercises as well– an instance of vicarious conditioned reinforcement. Still, from this experience the child may infer that the peer does not like math, and this may diminish the value of doing the exercise via imitative incentive learning. Nevertheless, despite the ambiguity and complexity of real-life situations, conditioned reinforcement and imitative incentive learning (alongside innate incentives) provide plausible explanations that can help speculate on the origin of people’s ends in their everyday life, and this sometimes may have practical benefits (e.g., during psychotherapy).

Based on our distinction between innate and acquired incentives, we have classified cognitive (or intrinsic) motives (e.g., those linked with novelty and curiosity; Chandler & Connell, 1987; Gottfried, 1983; Liquin & Lombrozo, 2020; Oudeyer & Kaplan, 2009; Ryan & Deci, 2000) as belonging to the former category. This choice was made because, historically, cognitive motives have been viewed as basic human needs (e.g., Maslow, 1943, 1954). However, a careful scrutiny reveals that this choice is debatable, and that intrinsic motives may be better interpreted as acquired, and not innate, incentives. This idea aligns with a recent perspective that views cognitive motives as emerging from information seeking during decision-making (Murayama, 2022; Murayama & Jach, 2025). According to this framework, agents compute the value associated with upcoming information, and if such value is deemed to be large, they initiate information-seeking behavior. Employing a decision-making framework, this idea can be modelled in terms of acquired incentives. For instance, when presented with a new toy, a child may attribute a large value to the toy for the simple reason that the toy is new. As the child familiarizes with the toy, the value associated with it (i.e., the novelty bonus) may progressively diminish. When presented for the first time, the toy can be interpreted as an acquired incentive to the extent that its value is not, so to speak, biologically determined, but it is dependent on the fact that the toy is new. A systematic analysis of cognitive motives goes beyond the scope of the present manuscript, but future literature may decide to consider cognitive motives as instances of acquired, and not innate, incentives, alongside conditioned reinforcement and imitative incentive learning.

We conclude our discussion by overviewing some interesting research avenues that our analysis opens up. First, note that important aspects of conditioned reinforcement and imitative incentive learning remain to be explored empirically. For instance, it remains to be investigated whether (and how) abstract concepts such as “being generous,” “respecting tradition,” or “being humble” can become acquired incentives via conditioned reinforcement. Moreover, a detailed empirical examination of extinction remains to be carried out in the context of conditioned reinforcement, especially to assess whether (and how) reinstatement occurs in this domain. In the domain of reinforcement learning research, how model-free and model-based algorithms interact during classical conditioning, thus engendering conditioned reinforcement, requires further exploration. Regarding imitative incentive learning, as documented above, the studies that have isolated this process above and beyond potential confounds are few, implying that various fundamental aspects remain to be elucidated. Examples concern the characteristics of the observed model (e.g., is a model who is more similar to oneself more likely to elicit imitative incentive learning?), the duration of the observation (e.g., is the strength of imitative incentive learning proportional to the duration of the observation?), and the role of extinction (e.g., does imitative incentive learning fade away if it is not supported by repeated observation? And does reinstatement play any role in imitative incentive learning?).

Besides encouraging research to explore conditioned reinforcement and imitative incentive learning further, our analysis raises interesting questions also at a broader level. One of such questions concerns the issue of how incentives are acquired during the lifespan (Bardi & Goodwin, 2011; Block & Block, 2006; Cieciuch et al., 2016; Fraley et al., 2012); Inglehart, 2018). For example, are children more susceptible to conditioned reinforcement and imitative incentive learning compared with adults? And is extinction of acquired incentives more likely in childhood compared with adulthood? Another domain where our analysis is relevant is that of cultural evolution. In the social sciences, there is growing interest in studying the mechanisms through which the culture of human populations evolves over history (Henrich, 2016; Mesoudi et al., 2006; Richerson & Boyd, 2008). Our analysis encourages scholars to extend this enquiry by exploring how the acquired incentives embraced by people within a population evolve over history (Rigoli & Lennon, 2025). Relatedly, a large body of research has investigated the link between certain social characteristics (e.g., economic affluence, economic inequality, and modernization) and people’s ethical values (Inglehart, 2018; Schwartz, 2006, 2013; Welzel, 2013; Welzel et al., 2003). Our analysis may contribute to this research by encouraging scholars to explore how the social structure promotes specific conditioned reinforcement and imitative incentive learning experiences which, in turn, may lead to the emergence of a specific set of ethical values within a community (Rigoli & Lennon, 2025).

In conclusion, by carrying out a critical analysis of available empirical evidence on conditioned reinforcement and social learning, our investigation offers a first examination of the question of how the reward function is learnt, in other words, of how people acquire their incentives during decision-making. This question, we argue, is central to the study of decision-making and yet has rarely been investigated in an explicit fashion so far. Our analysis pinpoints the specific aspects of this question that can be inferred based on available empirical evidence alongside the aspects that remain unknown and that are therefore open to future research.

## Data Availability

Not applicable.

## Footnotes

1 Although, when describing the transition function, the distinction between action and outcome is useful, this should not be viewed rigidly. An activity (e.g., running) can indeed be casted in terms of an outcome in the transition function (e.g., as expressed by the belief that if I get up early, then I can go running). In this way, the activity can be attributed a positive or negative number as prescribed by the reward function (e.g., I might attribute a positive number to running, reflecting the fact that I enjoy practicing this activity).

2 An outcome linked with positive or negative value may not only be an end, but, at the same time, also a means. Take a person who enjoys running as such. For this person, running may be at the same time an end, since it is enjoyable as such, as well as a means, since it is viewed as instrumental to achieve another end (e.g., being healthy).

3 Here, we use the term *end* and not the term *goal*. The reason is that the former term emphasizes finality—that is, it emphasizes that an outcome is valuable as such and not as a means for something else. The notion of goal is more ambiguous, as the scientific literature sometimes employs it in contexts where it is not clear whether a goal is valuable as such. Take a person instructed to perform a Stroop task (or, more generally, exposed to any taskset). The person is typically described as having the goal of reading the words while ignoring the colors. Though this is a perfectly legitimate use of the term goal, it begs the question of whether succeeding in reading the words is intrinsically valuable for the participant. Perhaps the participant does not attach any value to reading the words, but simply views this as means to achieve other ends such as feeling capable, avoiding boredom, being admired by the experimenter, or being compliant. Given such ambiguity that sometimes surrounds the term goal, here we prefer to use the term end.

4 The concept of innate reward and punishment as described here is virtually equivalent to the notion of unconditioned stimulus and to the similar notion of primary reinforcer as employed in the associative learning literature (Mackintosh, 1983; Pavlov, 1927).

5 Incentive sensitization theory (Robinson & Berridge, 2025) makes an interesting distinction between the concept of “wanting” and the concept of “liking,” the former capturing to what extent an animal is motivated to obtain an outcome (e.g., an addictive drug) and the latter indicating whether achieving the outcome (e.g., consuming the drug) is actually pleasurable. Although typically correlated, wanting and liking are viewed as distinct processes by incentive sensitization theory. The dissociation between the two, according to incentive sensitization theory, becomes particularly dramatic during an advanced stage of addiction, when drug consumption is experienced as little pleasurable despite the drug being desired intensely. The question of how the reward function is learnt, which is the focus of the present paper, concerns the notion of wanting and not that of liking, since wanting deals with motivation and decision-making.

6 A more parsimonious way to interpret the four quadrants taxonomy consists in postulating that the value attributed to an acquired incentive depends on the prediction error experienced during learning (Palminteri et al., 2015; Rigoli, 2019; Rigoli et al., 2016). In other words, the value may depend on whether, during learning, the acquired incentive signaled an outcome better or worse than expected. Within this logic, an acquired reward is established when a positive prediction error occurred, while an acquired punishment is established in the case of a negative prediction error.

7 In the context of reinforcement learning literature, it is important to clarify which models are relevant to explain conditioned reinforcement. As argued in the text, conditioned reinforcement is based on classical conditioning. This means that reinforcement learning models of classical conditioning are relevant (Dayan & Berridge, 2014; Sutton & Barto, 1999), while other models are not directly relevant. The latter include models dealing with learning the value of actions (e.g., *Q*-learning; Watkins & Dayan, 1992) as well as models dealing with planning (e.g., value iteration, policy iteration, or hierarchical reinforcement learning; Bellman, 1957; Botvinick et al., 2009; Sniedovich, 1991; Sutton & Barto, 1999).

8 We have just seen that our argument is that model-free learning is responsible for shaping the reward function. Does model-based learning also play a role in this process? We argue that this is not the case almost by definition. Indeed, the reward function concerns associations between outcomes and rewards, which is akin to the construct of cached value (acquired via model-free learning). It does not concern associations between stimuli and outcomes or between actions and outcomes, which instead concern model-based learning—therefore, model-based learning is excluded as a factor by definition.

9 Skinner’s idea has some resemblance with the construct of multifinality in social psychology (Kruglanski et al., 2015), occurring when a unique means is instrumental to achieve multiple goals. Despite some similarity, though, the concept of multifinality does not imply that the means becomes valuable in and of itself, but that its role remains that of a means.

10 Although acquired incentives and subgoals may be functionally similar, they are not equivalent. Ultimately, subgoals are treated by an agent as means to achieve something valuable, but they are not valuable in and of themselves. Therefore, they are not incentives. For example, a person may pursue the subgoal of becoming a businessman in order to become rich. Here the subgoal of becoming a businessman is not valuable as such, but only in virtue of being a means for becoming rich.

11 This form of imitation is often referred as emulation in the literature (e.g., Whiten et al., 2009).

12 The same confound applies to papers (Burke et al., 2010; Charpentier et al., 2020, 2024; Collette et al., 2017; Jern et al., 2017; Suzuki et al., 2012) that have used similar tasks as the one used by Najar and colleagues (2020).
